# A nomogram for predicting the risk of postoperative delirium in individuals undergoing cardiovascular surgery

**DOI:** 10.1111/ene.16483

**Published:** 2024-09-25

**Authors:** Chao Liu, Linfei Zhang, Weifeng Tang, Sheng Zhao, Mingke Li, Jinghang Li, Yongfeng Shao

**Affiliations:** ^1^ Department of Cardiac Surgery First Affiliated Hospital With Nanjing Medical University Nanjing China; ^2^ Department of Cardiothoracic Surgery Zhenjiang Clinical Medical College, Nanjing Medical University Zhenjiang China; ^3^ Department of Esophageal Surgery Nanjing Drum Tower Hospital, Affiliated Hospital of Medical School, Nanjing University Nanjing China

**Keywords:** cardiovascular surgery, postoperative delirium, predictive models, risk factors

## Abstract

**Background and Purpose:**

Delirium is a common mental disorder after adult cardiovascular surgery. Fifteen to 23% of patients undergoing cardiovascular surgery and cardiomyopathy experience delirium, and the efficacy of treatment interventions for delirium has been consistently unsatisfactory.

**Methods:**

A total of 729 patients who underwent cardiovascular surgery were randomly allocated into a training set and a validation set. A nomogram was developed using a logistic regression model to predict the incidence of delirium following cardiovascular surgery. The validity of the model was assessed by determining the receiver operating characteristic (ROC) curve, calculating the area under the ROC curve (AUROC), performing a calibration plot, and executing a decision curve analysis. This model was internally validated using the bootstrap method.

**Results:**

Postoperative delirium (POD) occurred in 165 cases (22.6%) among the 729 patients. Predictors included age, transient ischemic attack, length of preoperative stay, preoperative left ventricular injection fraction and N‐terminal pro‐B‐type natriuretic peptide level, and intraoperative infusion of dexmedetomidine and human fibrinogen. The nomogram showed sufficient differentiation and calibration (AUROC = 0.754, 95% confidence interval = 0.703–0.804). The calibration graphs showed that the predictive values of the nomogram were in agreement with the actual values. The analysis of the training and validation sets suggested that the model possessed specific clinical significance.

**Conclusions:**

In summary, the predictive model consists of seven factors that can roughly predict the occurrence of POD in patients who undergo cardiovascular surgery.

## INTRODUCTION

Delirium is a condition characterized by a rapid decline in attention, cognition, and overall mental condition, with no identifiable cause from existing neurocognitive disorders. The incidence might differ based on the individuals being observed and the choice of diagnostic instruments for delirium [1, 2]. The occurrence of delirium among patients undergoing cardiac surgery is approximately 15%–23% [3, 4, 5] and even could escalate to 50%–70% in patients with critical illness [6, 7].

Prior studies have shown a distinct correlation between delirium and multiple adverse consequences. In the short term, delirium has been found to substantially heighten morbidity and mortality rates, prolong hospital stays, and exacerbate the financial burden on patients. Furthermore, over the long term, delirium has been linked to increased postdischarge mortality, cognitive impairment, readmission rates, and development of dementia [8].

The etiological factors contributing to delirium are multifaceted. Age is an independent risk factor for the occurrence of delirium [9, 10, 11, 12, 13, 14], and mechanical ventilation is another risk factor for inducing delirium [15, 16, 17]. Atherosclerosis caused by peripheral vascular disease is also an independent predictive factor [6]. However, the underlying pathophysiological mechanisms remain unclear. Consequently, the efficacy of drug‐based prevention and treatment measures is limited. Previous studies have shown that implementing preventive interventions can effectively reduce the occurrence of delirium and enhance patient outcomes [18, 19, 20, 21]. Hence, the present study aims to examine preoperative and intraoperative factors that may influence the development of postoperative delirium (POD), with a focus on identifying the accurate predictors before the occurrence of delirium, so variables such as mechanical ventilation duration and intensive care unit (ICU) stay that cannot be determined prior to the onset of delirium were not in the scope of our study, then develop and validate a predictive model and generate a nomogram. Constructing a model for predicting POD and using it to immediately assess patients after surgery, screening high‐risk populations for preventive measures or early detection, could potentially yield positive outcomes.

## METHODS

### Study population

All the recruited subjects were of the Chinese Han population and from the Department of Cardiovascular Surgery at the First Affiliated Hospital of Nanjing Medical University. The research was conducted from January 2022 to December 2022, with all participants being duly informed about the study's objectives and providing their consent by signing a written form. The ethics review board of the First Affiliated Hospital of Nanjing Medical University granted approval for this study. The inclusion criteria for this study were as follows: (i) all patients were 18 years of age or older; and (ii) patients had undergone cardiovascular surgery, which included isolated procedures such as coronary artery bypass graft surgery (CABG), valve replacement (such as aortic valve replacement, mitral valve replacement, and tricuspid valve replacement), and valve repair surgery; combined procedures such as CABG combined with valve replacement or valvuloplasty; surgery for ascending aortic aneurysm or dissecting aneurysm; repair of atrial or ventricular defects; and other procedures (e.g., myxoma resection surgery). Exclusion criteria were as follows: (i) age < 18 years; (ii) preoperative history of dementia or cognitive impairment; (iii) nondirect vision surgery (such as aortic stent implantation, transcatheter aortic valve implantation surgery); (iv) cardiovascular surgery combined with other chest surgeries (such as lobectomy, esophageal cancer surgery); (v) preoperative coma, or coma or deep sedation within 3 days after surgery preventing subject from being evaluated; (vi) a second surgery within 3 days; (vii) death within 3 days after surgery; and (viii) case data missing.

### Assessment of delirium

We initially screened preoperative patients using the Mini‐Mental State Examination (MMSE) to exclude those with dementia. After the surgery, all participants were admitted to the ICU and given either propofol or dexmedetomidine to maintain a postoperative Richmond Agitation Sedation Scale (RASS) score between −2 and 0. As soon as the patient was conscious, evaluations were conducted every 8–12 h over three consecutive days by ICU nurses and doctors who had received relevant training. To determine whether a patient was suffering from POD, we utilized the Confusion of Assessment Method for the Intensive Care Unit (CAM‐ICU). Before initiating the assessment, the patient's baseline mental state was established, with the preceding 24‐h RASS score serving as a reference. If the RASS score was −4 or −5, the assessment was temporarily postponed; if the RASS score was higher than −4, further screening using the CAM‐ICU method was immediately performed.

### Intraoperative management

A standardized anesthesia protocol was typically employed. This involved the induction of anesthesia with either propofol or etomidate, in conjunction with fentanyl or sufentanil, and midazolam or remimazolam. Muscle relaxation was achieved using nondepolarizing muscle relaxants such as rocuronium or atracurium. Anesthesia was sustained through a combination of intravenous administration (propofol or dexmedetomidine) and inhalation anesthesia (sevoflurane, desflurane, or isoflurane). Invasive arterial blood pressure and continuous central vein monitoring (with or without Swan–Ganz pulmonary catheter) were used for hemodynamic monitoring. Intraoperative urinary output was monitored with catheterization. Cephalosporins were typically used as prophylactic antibiotics; vancomycin was administered in cases of penicillin allergy. Median sternotomy was the most common surgical approach, with a minority of patients undergoing minimally invasive right thoracotomy. Most surgeries were conducted under cardiopulmonary bypass (CPB), necessitating heparinization and achieving an activated clotting time (ACT) of ≥480 s prior to start CPB. Surgeries conducted without CPB also required heparinization and an ACT of >300 s. At the end of the procedure, heparin was neutralized with sulfated protamine. Depending on clinical conditions, bleeding, and heparin neutralization, vasoactive drugs, anticoagulants, blood transfusions, and blood products were administered during surgery.

### Statistical analysis

All participants were randomly divided into a training set (*n* = 513) and an internal validation set (*n* = 216) at a ratio of 7:3. The training set utilized univariate logistic regression analysis to identify potential predictors. Following this, a predictive model was developed using multivariate regression analysis. The model's predictors were evaluated via receiver operating characteristic (ROC) curves, and the model's discriminability was confirmed using calibration plots, with the bootstrap method employed for resampling validation. Subgroup analyses were also conducted. Moreover, decision curve analysis was used to assess the nomogram's practical utility in decision‐making.

For data analysis, we used R software (version 4.3.0), and a statistical significance threshold of *p* < 0.05 (bilateral) was applied.

## RESULTS

The study included a total of 729 participants with an average age of 59.6 ± 11.5 years, of whom 434 (59.5%) were male. Delirium occurred in 165 (22.6%) patients after surgery. There were statistically significant differences in age, hypertension, American Society of Anesthesiologists (ASA) physical status classification, transient ischemic attack (TIA), left ventricular injection fraction (LVEF), estimated glomerular filtration rate (eGFR), direct bilirubin, white blood cell count, N‐terminal pro‐B‐type natriuretic peptide (NT‐proBNP) level, type of surgery, duration of operation and CPB, clamp time, circulatory arrest, continuous dexmedetomidine infusion, intraoperative hemoglobin (Hb) level, red blood cell (RBC) transfusion, prothrombin complex concentrate and human fibrinogen infusion, and lactate level upon ICU admission after surgery (*p* < 0.05; TABLE S1).

Table S2 presents the listed attributes of the patients in both the training set and validation set. Generally, the two sets exhibit equilibrium and comparability (*p* > 0.05).

Univariate logistic regression analysis revealed that age, level of education, hypertension, ASA classification, cerebrovascular accident, TIA, length of preoperative hospital stay, LVEF, eGFR, NT‐proBNP level, duration of surgery and CPB, circulatory arrest, continuous dexmedetomidine infusion, propofol and γ‐aminobutyric acid administration, intraoperative Hb level, RBC transfusion, and infusion of prothrombin complex concentrate and human fibrinogen were potential predictors of POD (*p* < 0.1; Table 1).

**TABLE 1:** Univariate logistic regression analysis in training set.

Variables	*p*	OR (95% CI)
Preoperative		
Sex		
Male		1.00 (reference)
Female	0.469	0.86 (0.56–1.31)
Age, years	<0.001	1.04 (1.02–1.06)
BMI, kg/m^2^	0.356	1.03 (0.97–1.10)
Education		
Illiteracy		1.00 (reference)
Primary school	0.083	0.56 (0.29–1.08)
Junior middle school	0.240	0.72 (0.41–1.25)
High school	0.510	0.80 (0.41–1.56)
College	0.694	1.21 (0.47–3.09)
University and above	0.364	0.61 (0.21–1.77)
Smoking		
Never		1.00 (reference)
Yes	0.862	1.05 (0.63–1.73)
Ever	0.494	1.23 (0.68–2.20)
Drinking		
Never		1.00 (reference)
Yes	0.283	1.30 (0.80–2.10)
Hypertension		
No		1.00 (reference)
Yes	0.056	1.56 (0.99–2.46)
Diabetes		
No		1.00 (reference)
Yes	0.498	1.19 (0.72–1.95)
Asthma		
No		1.00 (reference)
Yes	0.259	1.90 (0.62–5.78)
Malignancy		
No		1.00 (reference)
Yes	0.995	1.00 (0.27–3.71)
ASA		
II + III		1.00 (reference)
IV	0.511	1.18 (0.72–1.95)
IIIE + IVE	<0.001	3.57 (1.82–6.99)
Cardiac surgery		
Never		1.00 (reference)
Ever	0.733	1.22 (0.38–3.92)
PCI		
Never		1.00 (reference)
Ever	0.584	0.73 (0.24–2.22)
CVA		
Never		1.00 (reference)
Ever	0.076	1.63 (0.95–2.81)
TIA		
Never		1.00 (reference)
Ever	0.031	2.52 (1.09–5.83)
AF		
Never		1.00 (reference)
Ever	0.196	1.35 (0.86–2.12)
Days before surgery	0.028	0.95 (0.90–0.99)
Statins		
No		1.00 (reference)
Yes	0.965	0.99 (0.58–1.69)
β‐blockers		
No		1.00 (reference)
Yes	0.273	0.74 (0.44–1.26)
Calcium channel blockers		
No		1.00 (reference)
Yes	0.977	0.99 (0.63–1.57)
ACEIs		
No		1.00 (reference)
Yes	0.349	0.59 (0.20–1.76)
ARBs		
No		1.00 (reference)
Yes	0.369	0.79 (0.47–1.33)
Diuretics		
No		1.00 (reference)
Yes	0.453	1.23 (0.71–2.13)
NSAIDs		
No		1.00 (reference)
Yes	0.615	1.14 (0.68–1.93)
LVEF, %	<0.001	0.96 (0.94–0.98)
eGFR, mL/min	0.004	0.99 (0.98–0.99)
ALT, U/L	0.116	0.99 (0.98–1.00)
AST, U/L	0.999	1.00 (0.99–1.01)
TG, mmol/L	0.436	0.88 (0.65–1.21)
CHOL, mmol/L	0.302	0.90 (0.75–1.09)
HDL, mmol/L	0.605	0.82 (0.40–1.71)
LDL, mmol/L	0.631	0.94 (0.72–1.22)
TB, μmol/L	0.340	1.01 (0.99–1.03)
DBIL, μmol/L	0.190	1.03 (0.99–1.07)
IBIL, μmol/L	0.687	1.01 (0.97–1.05)
WBC, 10^9^/L	0.116	1.06 (0.99–1.13)
NT‐ProBNP, pg/mL	0.045	1.01 (1.01–1.01)
Intraoperative		
Duration of surgery, h	0.003	1.19 (1.06–1.34)
Duration of CPB, h	0.045	1.17 (1.01–1.36)
Duration of clamp, min	0.192	1.00 (1.00–1.01)
Circulatory arrest		
No		1.00 (reference)
Yes	0.003	2.46 (1.36–4.45)
MAP, mmHg	0.186	0.98 (0.96–1.01)
Hb, g/L	0.027	0.86 (0.76–0.98)
Red blood cell transfusion	0.052	1.07 (1.00–1.14)
Medication administration		
Dexmedetomidine continuous infusion, mL/h	0.010	1.28 (1.06–1.55)
Propofol injection, mg	0.088	1.00 (1.00–1.01)
Aminocaproic acid injection, mg	0.039	1.11 (1.01–1.22)
Prothrombin complex concentrate infusion, IU	<0.001	1.01 (1.01–1.01)
Fibrinogen infusion, g	<0.001	1.44 (1.24–1.67)
Postoperative		
Lactate level, mmol/L	0.126	1.06 (0.98–1.15)

Abbreviations: ACEI, angiotensin converting enzyme inhibitor; AF, atrial fibrillation; ALT, alanine aminotransferase; ARB, angiotensin receptor blocker; ASA, American Society of Anesthesiologists; AST, aspartate aminotransferase; BMI, body mass index; CHOL, cholesterol; CI, confidence interval; CPB, cardiopulmonary bypass; CVA, cerebrovascular accident; DBIL, direct bilirubin; eGFR, estimated glomerular filtration rate; Hb, hemoglobin; HDL, high‐density lipoprotein; IBIL, Indirect bilirubin; LDL, low‐density lipoprotein; LVEF, left ventricular injection fraction; MAP, mean arterial pressure; NSAID, nonsteroidal anti‐inflammatory drug; NT‐ProBNP, N‐terminal pro‐B‐type natriuretic peptide; OR, odds ratio; PCI, percutaneous coronary intervention; TB, Total bilirubin; TG, triglyceride; TIA, transient ischemic attack; WBC, white blood cell count.

*Note*: AGE *p* < 0.001 indicates a statistically significant difference, with a 4% increase in the risk of POD for each additional year of age. ASA IIIE+IVE *p* < 0.001 indicates a statistically significant difference in ASA III and IV emergency surgery, with a 2.57 times increase in the risk of POD in reference to ASA II and III surgery. TIA Ever *p* = 0.031 indicates a statistically significant difference in patients who have had TIA in the past, with a 1.52 times increase in the risk of POD in reference to patients who never have TIA. Days before surgery *p* = 0.028 indicates a statistically significant difference, with a 5% decrease in the risk of POD for each additional day of hospital stay before surgery. LVEF *p* < 0.001 indicates a statistically significant difference, with a 4% decrease in the risk of POD for each additional unit of LVEF. eGFR *p* = 0.004 indicates a statistically significant difference, with a 1% decrease in the risk of POD for each additional unit of eGFR. NT‐ProBNP *p* = 0.045 indicates a statistically significant difference, with a 1% increase in the risk of POD for each additional unit of NT‐ProBNP. Duration of surgery *p* = 0.003 indicates a statistically significant difference, with a 19% increase in the risk of POD for each additional surgery hour. Duration of CPB *p* = 0.045 indicates a statistically significant difference, with a 17% increase in the risk of POD for each additional CPB hour. Circulatory arrest *p* = 0.003 indicates a statistically significant difference, with a 1.46 times higher in the risk of POD than patients who did not have circulatory arrest. Hb *p* = 0.027 indicates a statistically significant difference, with a 14% decrease in the risk of POD for each additional unit of Hb. Dexmedetomidine continuous infusion *p* = 0.010 indicates a statistically significant difference, with a 28% increase in the risk of POD for each additional unit usage of Dexmedetomidine. Aminocaproic acid injection *p* = 0.039 indicates a statistically significant difference, with a 11% increase in the risk of POD for each additional unit usage of Aminocaproic acid. Prothrombin complex concentrate infusion *p* < 0.001 indicates a statistically significant difference, with a 1% increase in the risk of POD for each additional unit usage of Prothrombin complex concentrate. Fibrinogen infusion *p* < 0.001 indicates a statistically significant difference, with a 44% increase in the risk of POD for each additional unit usage of human Fibrinogen.

Furthermore, a stepwise multivariate logistic regression analysis was conducted. The results indicated that with each additional year of age, the risk of POD increased by 0.06 times (*p* < 0.001, odds ratio [OR] = 1.06, 95% confidence interval [CI] = 1.04–1.09); having a TIA increased the risk of POD by 2.56 times (*p =* 0.006, OR = 3.56, 95% CI = 1.40–8.80); for each additional day of preoperative hospital stay, the risk of POD decreased by 0.06 times (*p* = 0.039, OR = 0.94, 95% CI = 0.89–1.00); for each unit increase in LVEF, the risk of POD decreased by 0.04 times (*p* = 0.002, OR = 0.96, 95% CI = 0.94–0.99); for each unit increase in NT‐proBNP level, the risk of POD increased by 0.00 times (*p* = 0.014, OR = 1.00, 95% CI = 1.00–1.00); for each unit increase in dexmedetomidine continuous infusion during surgery, the risk of POD increased by 0.39 times (*p* = 0.002, OR = 1.39, 95% CI = 1.12–1.72); and for each unit increase in human fibrinogen infusion, the risk of POD increased by 0.50 times (*p* < 0.001, OR = 1.50, 95% CI = 1.26–1.81; Table 2).

**TABLE 2:** Multivariate logistic regression analysis in training set.

Variables	β	*p*	OR (95% CI)
Age, years	0.06	<0.001	1.06 (1.04–1.09)
TIA			
No			1.00 (reference)
Yes	1.27	0.006	3.56 (1.40–8.80)
Days in hospital before surgery^a^	−0.06	0.039	0.94 (0.89–1.00)
LVEF, %	−0.04	0.002	0.96 (0.94–0.99)
NT‐proBNP, pg/mL^a^	0.00	0.014	1.00 (1.00–1.00)
Dexmedetomidine continuous infusion, mL/h	0.33	0.002	1.39 (1.12–1.72)
Fibrinogen infusion, g	0.41	<0.001	1.50 (1.26–1.81)

Abbreviations: CI, confidence interval; LVEF, left ventricular injection fraction; NT‐ProBNP, N‐terminal pro‐B‐type natriuretic peptide; OR, odds ratio; TIA, transient ischemic attack.

*Note*: AGE *p* < 0.001 indicates a statistically significant difference, with a 6% increase in the risk of POD for each additional year of age. IA Ever *p* = 0.006 indicates a statistically significant difference in patients who have had TIA in the past, with a 2.56 times increase in the risk of POD in reference to patients who never have TIA. Days before surgery *p* = 0.039 indicates a statistically significant difference, with a 6% decrease in the risk of POD for each additional day of hospital stay before surgery. LVEF *p* < 0.002 indicates a statistically significant difference, with a 4% decrease in the risk of POD for each additional unit of LVEF. NT‐ProBNP *p* = 0.014 indicates a statistically significant difference. Dexmedetomidine continuous infusion *p* = 0.002 indicates a statistically significant difference, with a 39% increase in the risk of POD for each additional unit usage of Dexmedetomidine. Fibrinogen infusion *p* < 0.001 indicates a statistically significant difference, with a 50% increase in the risk of POD for each additional unit usage of human Fibrinogen.

^a^ The OR values are close to 1, but the *p*‐values are significant, and the CIs do not actually include 1. Therefore, both predictive factors still have statistical significance.

**FIGURE 1:**
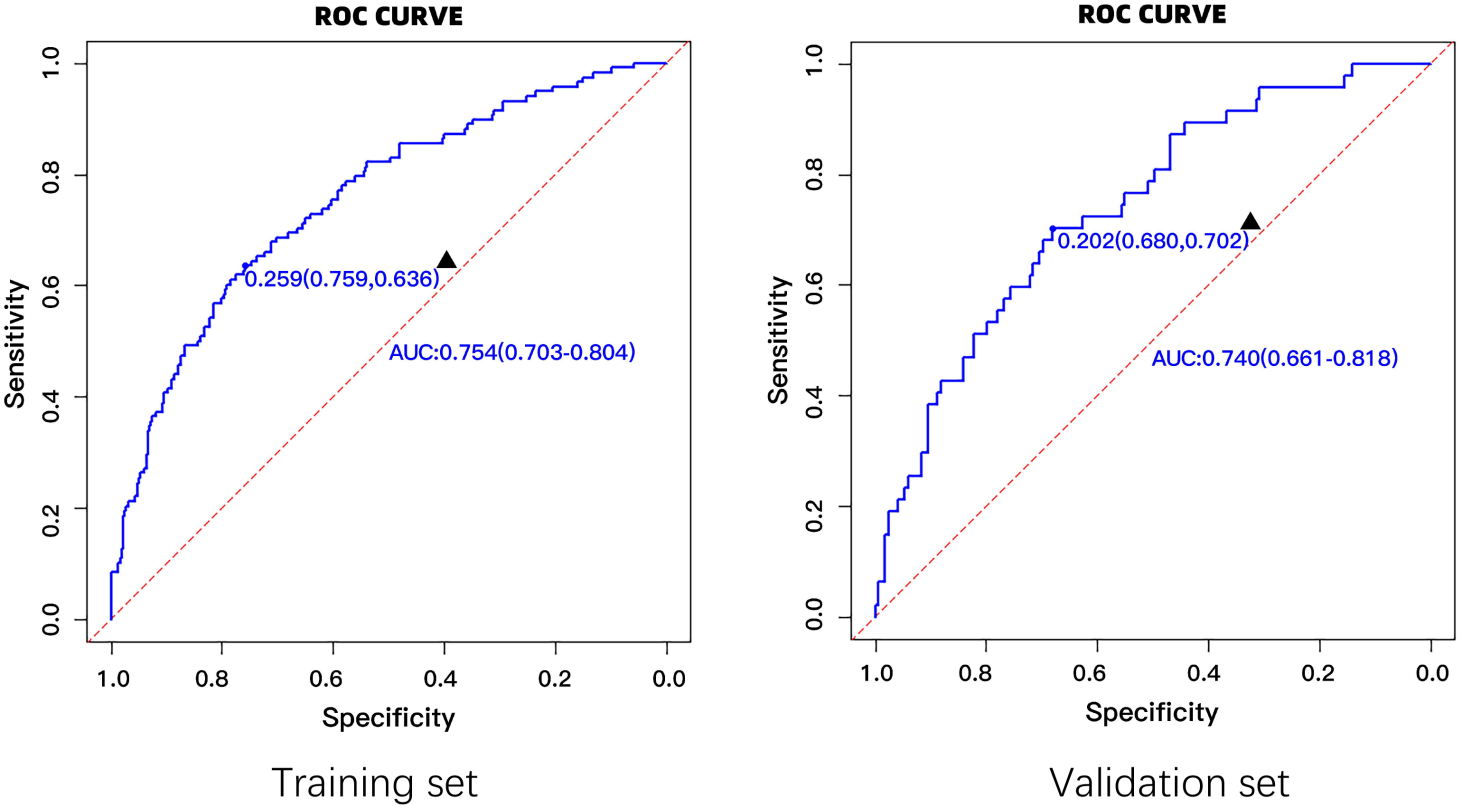
Receiver operating characteristic curve in training and validation sets. *According to the optimal cutoff value calculated by the Youden index and the corresponding sensitivity and specificity. AUC, area under the curve.

The area under the ROC curve (AUROC) for the training set was found to be 0.754 (95% CI = 0.703–0.804). Similarly, the area under the curve for the internal validation set was found to be 0.740 (95% CI = 0.661–0.818), indicating that the model exhibits promising predictive capabilities (Figure 1).

**FIGURE 2:**
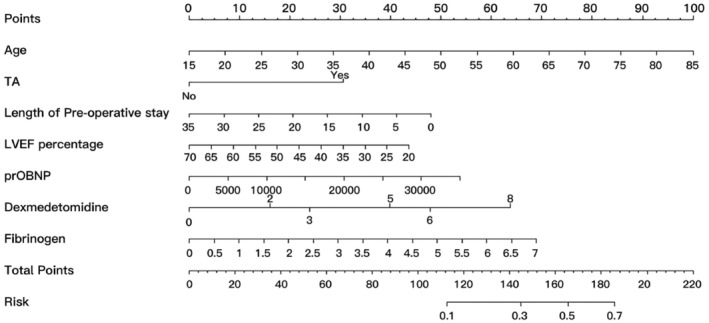
Nomogram for predicting postoperative delirium. LVEF, left ventricular injection fraction; proBNP, pro‐B‐type natriuretic peptide; TIA, transient ischemic attack.

Our study identified seven predictive factors for POD: age, TIA, length of preoperative stays, preoperative LVEF and NT‐proBNP level, and intraoperative dexmedetomidine and human fibrinogen infusion. Each factor was assigned a numerical value, and the total score was calculated by adding up these individual scores. This total score served as an indicator of the predicted risk of POD (Figure 2).

**FIGURE 3:**
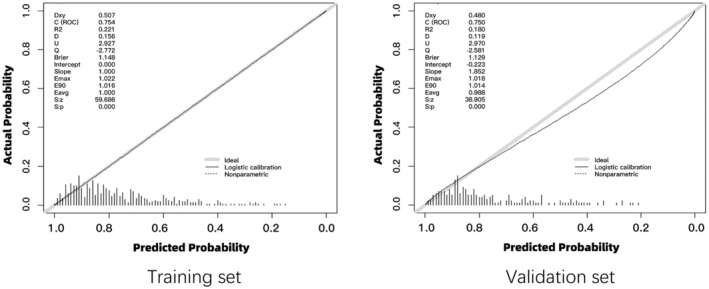
Calibration plot of actual versus predicted postoperative delirium in training and validation sets. Brier, brier score; C, index of concordance; D, discrimination index; Dxy, Somers' D; E90, 90th percentile of error; Eavg, expected average difference; Emax, maximum absolute error; Q, quality index; R2, Nagelkerke‐Cox‐Snell‐Maddala‐Magee R‐squared index; S:p, Spiegelhalter *Z*‐test *p* value; S:z, Spiegelhalter *Z*‐test *z* value; U, unreliability index.

The calibration curves for both the training and validation sets demonstrated a strong concordance between the predicted values of the nomogram and the observed ones, thereby affirming the high accuracy of the predictive model (Figure 3).

**FIGURE 4:**
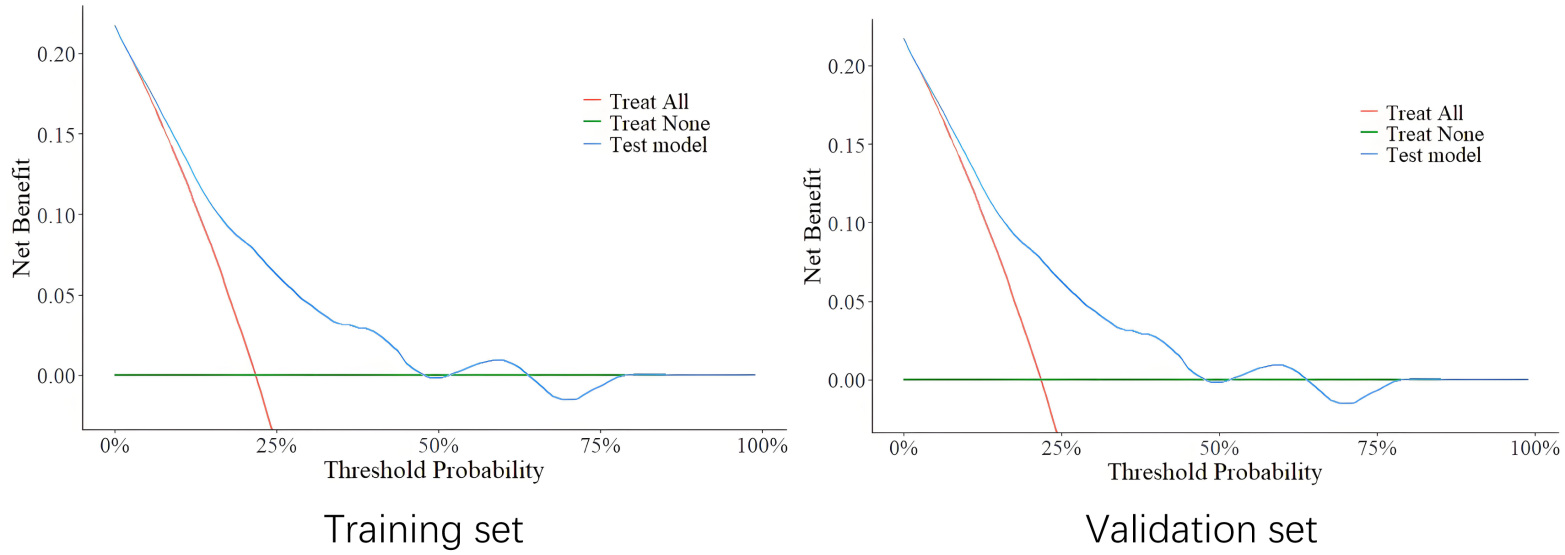
Decision curve analysis in training and validation sets.

The analysis of the training and validation sets implied that the model possessed specific clinical significance (Figure 4).

A subgroup analysis was conducted, categorizing the subgroups based on circulatory arrest status and type of surgery. The AUROC for the subgroup without circulatory arrest was 0.79 (95% CI = 0.51–1.00); for the subgroup with circulatory arrest, it was 0.73 (95% CI = 0.59–0.87). In the group of patients who underwent valve replacement or repair, the AUROC was found to be 0.91 (95% CI = 0.75–1.00). In the off‐pump CABG group, the AUROC was 0.58 (95% CI = 0.31–0.86). The on‐pump CABG group had an AUROC of 0.80 (95% CI = 0.60–1.00). For the subgroup of CABG combined with valve replacement or repair, the AUROC was 0.75, with a 95% CI of 0.70–0.80. Finally, patients who underwent surgery for ascending aortic aneurysm or aortic dissection had an AUROC of 0.74 (95% CI = 0.66–0.82; Table 3).

**TABLE 3:** Stratified analysis in training and test sets by cardiopulmonary bypass arrest status and surgery type.

Data	AUROC (95% CI)	Accuracy (95% CI)	Sensitivity (95% CI)	Specificity (95% CI)	PPV (95% CI)	NPV (95% CI)
Train	0.75 (0.70–0.80)	0.73 (0.69–0.77)	0.76 (0.72–0.80)	0.64 (0.55–0.72)	0.44 (0.37–0.52)	0.88 (0.84–0.91)
Test	0.74 (0.66–0.82)	0.73 (0.66–0.79)	0.78 (0.72–0.84)	0.53 (0.39–0.68)	0.40 (0.28–0.53)	0.86 (0.80–0.91)
Circulatory arrest						
No	0.79 (0.51–1.00)	0.79 (0.54–0.94)	0.57 (0.21–0.94)	0.92 (0.76–1.00)	0.79 (0.57–1.00)	0.80 (0.45–1.00)
Yes	0.73 (0.59–0.87)	0.75 (0.65–0.83)	0.78 (0.69–0.87)	0.53 (0.28–0.79)	0.31 (0.13–0.49)	0.90 (0.83–0.97)
Procedure						
Isolated						
Valve replacement	0.91 (0.75–1.00)	0.80 (0.52–0.96)	0.91 (0.74–1.00)	0.50 (0.01–0.99)	0.67 (0.13–1.00)	0.83 (0.62–1.00)
CABG, off‐pump	0.58 (0.31–0.86)	0.60 (0.36–0.81)	0.62 (0.35–0.88)	0.57 (0.21–0.94)	0.44 (0.12–0.77)	0.73 (0.46–0.99)
CABG, on‐pump	0.80 (0.60–0.99)	0.65 (0.43–0.84)	0.42 (0.14–0.70)	0.91 (0.74–1.00)	0.59 (0.35–0.82)	0.83 (0.54–1.00)
Combined	0.75 (0.70–0.80)	0.73 (0.69–0.77)	0.76 (0.72–0.80)	0.64 (0.55–0.72)	0.44 (0.37–0.52)	0.88 (0.84–0.91)
Ascending aortic aneurysm or dissection surgery	0.74 (0.66–0.82)	0.73 (0.66–0.79)	0.78 (0.72–0.84)	0.53 (0.39–0.68)	0.40 (0.28–0.53)	0.86 (0.80–0.91)

Abbreviations: AUROC, area under the receiver operating characteristic curve; CABG, coronary artery bypass graft surgery; CI, confidence interval; NPV, negative predictive value; PPV, positive predictive value.

## DISCUSSION

Various negative consequences have been linked to delirium, encompassing immediate impacts like falls, aspiration pneumonia, heightened susceptibility to surgical infections, cerebral vascular accidents, pressure ulcers, and additional complications [22, 23]. It also has been associated with extended durations in the ICU and medical facility [24], elevated rates of short‐term death, and escalated expenses for hospitalization [9, 25, 26, 27]. Moreover, delirium has been shown to have negative long‐term consequences, such as a decline in cognitive function over time in patients [28, 29, 30, 31] as well as increased 1‐year mortality rate and disability [32, 33].

The etiologies of POD can be classified as predisposing and precipitating factors. Predisposing risk factors include advanced age (≥65 years old), cognitive impairment, frailty, comorbidities (e.g., cardiovascular and kidney diseases), depression or other mental illnesses [34, 35], alcoholism, malnutrition [36, 37], visual and hearing impairment [9, 38], and peripheral vascular diseases [6] among others. The precipitating factors include sudden illnesses (like sepsis, hypoglycemia, stroke, and liver failure), trauma (such as fractures, head injuries), surgery, anesthesia, CPB duration, intraoperative massive blood loss [39], dehydration, psychological stress [9, 40], blood poverty, abnormal plasma protein levels, hypoxia, pain [41, 42], long‐term immobilization, sleep disorders [43], et cetera. The overall risk was contingent upon the quantity and intensity of individual risk factors. Generally, multiple precipitating factors could affect patients at the same time. Furthermore, the occurrence of delirium was also linked to the utilization, withdraw, and modifications of medication.

Age is an independent risk factor for the occurrence of delirium [9, 10, 11, 12, 13, 14], and our research showed that for each additional year of age, the risk of POD increased by 6%.

On the one hand, patients exhibiting cognitive decline or dementia were at a heightened risk of developing POD [44, 45, 46]. Rudolph et al. [42] identified four independent predictors for POD: prior stroke or transient ischemic attack (TIA), MMSE score, abnormal serum albumin, and the Geriatric Depression Scale. TIA was also recognized in our model. Another predictive model for POD, devised by Varga‐Martínez et al. [47], similarly incorporated the MMSE score. However, the predictors in both these models were confined to preoperative variables and did not consider the influence of intraoperative variables such as surgical procedures and anesthesia on POD. Our model has precisely addressed this gap. On the other hand, episodes of POD could also accelerate the progression of symptoms in patients suffering from dementia or cognitive dysfunction [48]. We preoperatively screened out patients with dementia (severe cognitive impairment) using the MMSE. Yet, we neither categorized cognitive dysfunction nor excluded mild cognitive decline, which are areas that require enhancement in our future research.

Our study discovered that intraoperative administration of human fibrinogen could significantly increase the risk of POD, which may be attributed to intraoperative blood loss. Notably, existing literature does not support our findings, prompting us to further investigate the correlation between intraoperative human fibrinogen usage and POD. Additionally, we observed a 39% increase in POD risk in patients administered with dexmedetomidine intraoperatively. This aligns with a randomized placebo‐controlled trial demonstrating a 47% increase in POD in patients who received dexmedetomidine during cardiac surgery with CPB and within 24 h postoperation, potentially linked to hypotension that the drug provoked [49]. However, a contrasting systematic review and meta‐analysis revealed that dexmedetomidine could reduce the risk of POD in patients aged >65 years undergoing noncardiac surgery. For those younger than 65 years, no correlation was found between dexmedetomidine use and POD risk [50]. However, almost all patients were administered postoperative analgesics such as fentanyl, sufentanil, or morphine in these included study, which could potentially impact the final outcomes.

Numerous prior studies have affirmed the effectiveness of preventive intervention in reducing the incidence of delirium and improving prognosis [18, 19, 20, 21]. Consequently, it is imperative to develop a delirium prediction model that can swiftly identify high‐risk groups and facilitate the prevention or prompt detection of delirium. Despite the significant attention dedicated to predictive models for delirium following cardiac surgery in existing literature, certain constraints remain, such as limited predictive power, insufficient sample size, potential bias, and lack of visual predictive models [14, 46, 51, 52]. Our study pinpointed seven predictors for POD: age, TIA, preoperative hospital stays, preoperative LVEF and NT‐proBNP level, and intraoperative dexmedetomidine and human fibrinogen infusion. All these factors could be identified prior to the onset of POD. A predictive model was developed with these variables, followed by the creation of a nomogram to visually represent the model and evaluate the probability of POD. Upon internal validation, the model demonstrated remarkable discrimination, calibration, and clinical utility.

## LIMITATIONS

First, all the subjects recruited for this study were from a single center, resulting in the predictive model not being externally validated and potentially introducing selection bias. Second, we only screened out patients with dementia and severe cognitive impairment using the MMSE, overlooking the potential subtle effects of cognitive decline. In future studies, these significant confounding factors should be taken into account. Moreover, we plan to investigate the correlation between intraoperative administration of human fibrinogen and the onset of POD, aiming to unravel the possible underlying mechanisms.

## AUTHOR CONTRIBUTIONS

**Chao Liu:** Conceptualization; investigation; writing – original draft; formal analysis; data curation; methodology. **Linfei Zhang:** Data curation. **Weifeng Tang:** Writing – original draft. **Sheng Zhao:** Writing – original draft. **Mingke Li:** Data curation. **Jinghang Li:** Data curation. **Yongfeng Shao:** Supervision; validation; resources.

## CONFLICT OF INTEREST STATEMENT

The authors declare no conflict of interest.

## ETHICS STATEMENT

All procedures performed in this study involving human participants were in accordance with ethical standards and approved by the ethics committee of the First Affiliated Hospital with Nanjing Medical University.

## CONSENT STATEMENT

All participants were duly informed about the objectives of the study and provided their consent by signing a written form.

## Supporting information


Table S1.

Table S2.

## Data Availability

The research data supporting this publication are available in the online supplementary material.

## References

[1] Sockalingam S , Parekh N , Bogoch II , et al. Delirium in the postoperative cardiac patient: a review. J Card Surg. 2005;20(6):560‐567. 16309412 10.1111/j.1540-8191.2005.00134.x

[2] Yokota H , Ogawa S , Kurokawa A , Yamamoto Y . Regional cerebral blood flow in delirium patients. Psychiatry Clin Neurosci. 2003;57(3):337‐339. 12753576 10.1046/j.1440-1819.2003.01126.x

[3] Gosselt AN , Slooter AJ , Boere PR , Zaal IJ . Risk factors for delirium after on‐pump cardiac surgery: a systematic review. Crit Care. 2015;19(1):346. 26395253 10.1186/s13054-015-1060-0 PMC4579578

[4] Aitken SJ , Blyth FM , Naganathan V . Incidence, prognostic factors and impact of postoperative delirium after major vascular surgery: a meta‐analysis and systematic review. Vasc Med. 2017;22(5):387‐397. 28784053 10.1177/1358863X17721639

[5] Oldroyd C , Scholz AFM , Hinchliffe RJ , McCarthy K , Hewitt J , Quinn TJ . A systematic review and meta‐analysis of factors for delirium in vascular surgical patients. J Vasc Surg. 2017;66(4):1269‐1279.e9. 28942855 10.1016/j.jvs.2017.04.077

[6] Norkiene I , Ringaitiene D , Misiuriene I , et al. Incidence and precipitating factors of delirium after coronary artery bypass grafting. Scand Cardiovasc J. 2007;41(3):180‐185. 17487768 10.1080/14017430701302490

[7] Mcpherson JA , Wagner CE , Boehm LM , et al. Delirium in the cardiovascular ICU: exploring modifiable risk factors. Crit Care Med. 2013;41(2):405‐413. 23263581 10.1097/CCM.0b013e31826ab49b PMC3557701

[8] Witlox J , Eurelings LS , de Jonghe JF , Kalisvaart KJ , Eikelenboom P , van Gool WA . Delirium in elderly patients and the risk of postdischarge mortality, institutionalization, and dementia: a meta‐analysis. JAMA. 2010;304(4):443‐451. 20664045 10.1001/jama.2010.1013

[9] Inouye SK , Westendorp RG , Saczynski JS . Delirium in elderly people. Lancet. 2014;383(9920):911‐922. 23992774 10.1016/S0140-6736(13)60688-1 PMC4120864

[10] Bucerius J , Gummert JF , Borger MA , et al. Predictors of delirium after cardiac surgery delirium: effect of beating‐heart (off‐pump) surgery. J Thorac Cardiovasc Surg. 2004;127(1):57‐64. 14752413 10.1016/s0022-5223(03)01281-9

[11] Van Der Mast RC , Van Den Broek WW , Fekkes D , Pepplinkhuizen L , Habbema JD . Incidence of and preoperative predictors for delirium after cardiac surgery. J Psychosom Res. 1999;46(5):479‐483. 10404482 10.1016/s0022-3999(99)00002-1

[12] Giltay EJ , Huijskes RV , Kho KH , Blansjaar BA , Rosseel PM . Psychotic symptoms in patients undergoing coronary artery bypass grafting and heart valve operation. Eur J Cardiothorac Surg. 2006;30(1):140‐147. 16723244 10.1016/j.ejcts.2006.03.056

[13] Santos FS , Velasco IT , Fraguas R Jr . Risk factors for delirium in the elderly after coronary artery bypass graft surgery. Int Psychogeriatr. 2004;16(2):175‐193. 15318763

[14] Afonso A , Scurlock C , Reich D , et al. Predictive model for postoperative delirium in cardiac surgical patients. Semin Cardiothorac Vasc Anesth. 2010;14(3):212‐217. 20647262 10.1177/1089253210374650

[15] Van Rompaey B , Elseviers MM , Schuurmans MJ , Shortridge‐Baggett LM , Truijen S , Bossaert L . Risk factors for delirium in intensive care patients: a prospective cohort study. Crit Care. 2009;13(3):R77. 19457226 10.1186/cc7892 PMC2717440

[16] Shehabi Y , Riker RR , Bokesch PM , et al. Delirium duration and mortality in lightly sedated, mechanically ventilated intensive care patients. Crit Care Med. 2010;38(12):2311‐2318. 20838332 10.1097/CCM.0b013e3181f85759

[17] Almeida IC , Soares M , Bozza FA , et al. The impact of acute brain dysfunction in the outcomes of mechanically ventilated cancer patients. PLoS One. 2014;9(1):e85332. 24465538 10.1371/journal.pone.0085332 PMC3899009

[18] Young J , Murthy L , Westby M , Akunne A , O'Mahony R , Guideline Development Group . Diagnosis, prevention, and management of delirium: summary of NICE guidance. BMJ. 2010;341:c3704. 20667955 10.1136/bmj.c3704

[19] Inouye SK , Bogardus ST Jr , Charpentier PA , et al. A multicomponent intervention to prevent delirium in hospitalized older patients. N Engl J Med. 1999;340(9):669‐676. 10053175 10.1056/NEJM199903043400901

[20] Kalisvaart KJ , De Jonghe JF , Bogaards MJ , et al. Haloperidol prophylaxis for elderly hip‐surgery patients at risk for delirium: a randomized placebo‐controlled study. J Am Geriatr Soc. 2005;53(10):1658‐1666. 16181163 10.1111/j.1532-5415.2005.53503.x

[21] Katznelson R , Djaiani GN , Borger MA , et al. Preoperative use of statins is associated with reduced early delirium rates after cardiac surgery. Anesthesiology. 2009;110(1):67‐73. 19104172 10.1097/ALN.0b013e318190b4d9

[22] Guthrie PF , Rayborn S , Butcher HK . Evidence‐based practice guideline: delirium. J Gerontol Nurs. 2018;44(2):14‐24. 29378075 10.3928/00989134-20180110-04

[23] Martin BJ , Buth KJ , Arora RC , Baskett RJ . Delirium as a predictor of sepsis in post‐coronary artery bypass grafting patients: a retrospective cohort study. Crit Care. 2010;14(5):R171. 20875113 10.1186/cc9273 PMC3219273

[24] Brown CHT , Laflam A , Max L , et al. The impact of delirium after cardiac surgical procedures on postoperative resource use. Ann Thorac Surg. 2016;101(5):1663‐1669. 27041454 10.1016/j.athoracsur.2015.12.074 PMC5406132

[25] Geriatric Medicine Research Collaborative . Delirium is prevalent in older hospital inpatients and associated with adverse outcomes: results of a prospective multi‐centre study on world delirium awareness day. BMC Med. 2019;17(1):229. 31837711 10.1186/s12916-019-1458-7 PMC6911703

[26] Ely EW , Shintani A , Truman B , et al. Delirium as a predictor of mortality in mechanically ventilated patients in the intensive care unit. JAMA. 2004;291(14):1753‐1762. 15082703 10.1001/jama.291.14.1753

[27] Israni J , Lesser A , Kent T , Ko K . Delirium as a predictor of mortality in US Medicare beneficiaries discharged from the emergency department: a national claims‐level analysis up to 12 months. BMJ Open. 2018;8(5):e021258. 10.1136/bmjopen-2017-021258 PMC5942463 29730630

[28] Pandharipande PP , Girard TD , Ely EW . Long‐term cognitive impairment after critical illness. N Engl J Med. 2014;370(2):185‐186. 10.1056/NEJMc1313886 24401069

[29] Lin SM , Liu CY , Wang CH , et al. The impact of delirium on the survival of mechanically ventilated patients. Crit Care Med. 2004;32(11):2254‐2259. 15640638 10.1097/01.ccm.0000145587.16421.bb

[30] Saczynski JS , Marcantonio ER , Quach L , et al. Cognitive trajectories after postoperative delirium. N Engl J Med. 2012;367(1):30‐39. 22762316 10.1056/NEJMoa1112923 PMC3433229

[31] Kunicki ZJ , Ngo LH , Marcantonio ER , et al. Six‐year cognitive trajectory in older adults following major surgery and delirium. JAMA Intern Med. 2023;183(5):442‐450. 36939716 10.1001/jamainternmed.2023.0144 PMC10028541

[32] Pisani MA , Kong SY , Kasl SV , Murphy TE , Araujo KL , Van Ness PH . Days of delirium are associated with 1‐year mortality in an older intensive care unit population. Am J Respir Crit Care Med. 2009;180(11):1092‐1097. 19745202 10.1164/rccm.200904-0537OC PMC2784414

[33] Altman MT , Knauert MP , Murphy TE , Ahasic AM , Chauhan Z , Pisani MA . Association of intensive care unit delirium with sleep disturbance and functional disability after critical illness: an observational cohort study. Ann Intensive Care. 2018;8(1):63. 29740704 10.1186/s13613-018-0408-4 PMC5940933

[34] Smith PJ , Attix DK , Weldon BC , Greene NH , Monk TG . Executive function and depression as independent risk factors for postoperative delirium. Anesthesiology. 2009;110(4):781‐787. 19326492 10.1097/aln.0b013e31819b5bc2 PMC2757787

[35] Wilson K , Broadhurst C , Diver M , Jackson M , Mottram P . Plasma insulin growth factor‐1 and incident delirium in older people. Int J Geriatr Psychiatry. 2005;20(2):154‐159. 15660412 10.1002/gps.1265

[36] Velayati A , Vahdat Shariatpanahi M , Shahbazi E , Vahdat Shariatpanahi Z . Association between preoperative nutritional status and postoperative delirium in individuals with coronary artery bypass graft surgery: a prospective cohort study. Nutrition. 2019;66:227‐232. 31357095 10.1016/j.nut.2019.06.006

[37] Sanford AM , Flaherty JH . Do nutrients play a role in delirium? Curr Opin Clin Nutr Metab Care. 2014;17(1):45‐50. 24296414 10.1097/MCO.0000000000000022

[38] Smith TO , Cooper A , Peryer G , Griffiths R , Fox C , Cross J . Factors predicting incidence of post‐operative delirium in older people following hip fracture surgery: a systematic review and meta‐analysis. Int J Geriatr Psychiatry. 2017;32(4):386‐396. 28093812 10.1002/gps.4655

[39] Bohner H , Hummel TC , Habel U , et al. Predicting delirium after vascular surgery: a model based on pre‐ and intraoperative data. Ann Surg. 2003;238(1):149‐156. 12832977 10.1097/01.sla.0000077920.38307.5f PMC1422662

[40] Marcantonio ER . Delirium in hospitalized older adults. N Engl J Med. 2017;377(15):1456‐1466. 29020579 10.1056/NEJMcp1605501 PMC5706782

[41] National Institute for Health and Clinical Excellence . Delirium: diagnosis, prevention and management. Royal College of Physicians; 2010 Accessed at www.niceorguk/CG103 and https://wwwncbinlmnihgov/pubmed/22319805 22319805

[42] Rudolph JL , Jones RN , Levkoff SE , et al. Derivation and validation of a preoperative prediction rule for delirium after cardiac surgery. Circulation. 2009;119(2):229‐236. 19118253 10.1161/CIRCULATIONAHA.108.795260 PMC2735244

[43] Gabor JY , Cooper AB , Crombach SA , et al. Contribution of the intensive care unit environment to sleep disruption in mechanically ventilated patients and healthy subjects. Am J Respir Crit Care Med. 2003;167(5):708‐715. 12598213 10.1164/rccm.2201090

[44] Jin Z , Hu J , Ma D . Postoperative delirium: perioperative assessment, risk reduction, and management. Br J Anaesth. 2020;125(4):492‐504. 32798069 10.1016/j.bja.2020.06.063

[45] Kim EM , Li G , Kim M . Development of a risk score to predict postoperative delirium in patients with hip fracture. Anesth Analg. 2020;130(1):79‐86. 31478933 10.1213/ANE.0000000000004386 PMC6917900

[46] Bakker RC , Osse RJ , Tulen JH , Kappetein AP , Bogers AJ . Preoperative and operative predictors of delirium after cardiac surgery in elderly patients. Eur J Cardiothorac Surg. 2012;41(3):544‐549. 22345177 10.1093/ejcts/ezr031

[47] De La Varga‐Martínez O , Gomez‐Pesquera E , Munoz‐Moreno MF , et al. Development and validation of a delirium risk prediction preoperative model for cardiac surgery patients (DELIPRECAS): an observational multicentre study. J Clin Anesth. 2021;69:110158. 33296785 10.1016/j.jclinane.2020.110158

[48] Fong TG , Jones RN , Shi P , et al. Delirium accelerates cognitive decline in Alzheimer disease. Neurology. 2009;72(18):1570‐1575. 19414723 10.1212/WNL.0b013e3181a4129a PMC2677515

[49] Turan A , Duncan A , Leung S , et al. Dexmedetomidine for reduction of atrial fibrillation and delirium after cardiac surgery (DECADE): a randomised placebo‐controlled trial. Lancet. 2020;396(10245):177‐185. 32682483 10.1016/S0140-6736(20)30631-0

[50] Qin C , Jiang Y , Lin C , Li A , Liu J . Perioperative dexmedetomidine administration to prevent delirium in adults after non‐cardiac surgery: a systematic review and meta‐analysis. J Clin Anesth. 2021;73:110308. 33930679 10.1016/j.jclinane.2021.110308

[51] Lindroth H , Bratzke L , Purvis S , et al. Systematic review of prediction models for delirium in the older adult inpatient. BMJ Open. 2018;8(4):e019223. 10.1136/bmjopen-2017-019223 PMC5931306 29705752

[52] Cai S , Li J , Gao J , Pan W , Zhang Y . Prediction models for postoperative delirium after cardiac surgery: systematic review and critical appraisal. Int J Nurs Stud. 2022;136:104340. 36208541 10.1016/j.ijnurstu.2022.104340

