# Supplementary material for: A nomogram for predicting the risk of postoperative delirium in individuals undergoing cardiovascular surgery
Source: Eur J Neurol. 2024 Sep 25;31(12):e16483. doi: 10.1111/ene.16483 (PMC11555157; doi:10.1111/ene.16483)
Supplement: Supplementary file 1 — Table S1. [file ENE-31-e16483-s001.pdf]

Supplementary table 1 Characteristics of patients

Variable	Total (n = 729)	Delirium		Statistic	*P*
		No (n = 564)	Yes (n = 165)		
**Preoperative**					
Sex, n (%)				χ^2=0.462	0.497
Male	434 (59.53)	332 (58.87)	102 (61.82)		
Female	295 (40.47)	232 (41.13)	63 (38.18)		
Age (y), Mean ± SD	59.61 ± 11.49	58.47 ± 11.70	63.52 ± 9.77	t=-5.052	**<.001**
BMI (kg/m^2), Mean ± SD	24.29 ± 3.39	24.21 ± 3.47	24.56 ± 3.08	t=-1.195	0.233
Education, n (%)				χ^2=6.805	0.236
Illiteracy	160 (21.95)	114 (20.21)	46 (27.88)		
Primary school	142 (19.48)	115 (20.39)	27 (16.36)		
Junior middle school	243 (33.33)	196 (34.75)	47 (28.48)		
High school	106 (14.54)	82 (14.54)	24 (14.55)		
College	37 (5.08)	27 (4.79)	10 (6.06)		
University and above	41 (5.62)	30 (5.32)	11 (6.67)		
Smoking, n (%)				χ^2=0.900	0.638
Never	451 (61.87)	352 (62.41)	99 (60.00)		
Yes	171 (23.46)	133 (23.58)	38 (23.03)		
Ever	107 (14.68)	79 (14.01)	28 (16.97)		
Drinking, n (%)				χ^2=2.279	0.131
Never	566 (77.64)	445 (78.90)	121 (73.33)		
Yes	163 (22.36)	119 (21.10)	44 (26.67)		
Hypertension, n (%)				χ^2=10.243	**0.001**
No	248 (34.02)	209 (37.06)	39 (23.64)		
Yes	481 (65.98)	355 (62.94)	126 (76.36)		
Diabetes, n (%)				χ^2=0.981	0.322
No	581 (79.7)	454 (80.50)	127 (76.97)		
Yes	148 (20.3)	110 (19.50)	38 (23.03)		
Asthma, n (%)				χ^2=0.939	0.333
No	712 (97.67)	553 (98.05)	159 (96.36)		
Yes	17 (2.33)	11 (1.95)	6 (3.64)		
Malignancy, n (%)				χ^2=1.143	0.285
No	709 (97.26)	551 (97.70)	158 (95.76)		
Yes	20 (2.74)	13 (2.30)	7 (4.24)		
ASA, n (%)				χ^2=30.037	**<.001**
II+III	496 (68.04)	398 (70.57)	98 (59.39)		
IV	180 (24.69)	141 (25.00)	39 (23.64)		
IIIE+IVE	53 (7.27)	25 (4.43)	28 (16.97)		
Cardiac surgery, n (%)				χ^2=0.012	0.912
Never	710 (97.39)	550 (97.52)	160 (96.97)		
Ever	19 (2.61)	14 (2.48)	5 (3.03)		

PCI, n (%)				χ^2=0.506	0.477
Never	695 (95.34)	536 (95.04)	159 (96.36)		
Ever	34 (4.66)	28 (4.96)	6 (3.64)		
CVA, n (%)				χ^2=2.280	0.131
Never	631 (86.56)	494 (87.59)	137 (83.03)		
Ever	98 (13.44)	70 (12.41)	28 (16.97)		
TIA, n (%)				χ^2=4.778	**0.029**
Never	698 (95.75)	545 (96.63)	153 (92.73)		
Ever	31 (4.25)	19 (3.37)	12 (7.27)		
AF, n (%)				χ^2=3.493	0.062
Never	536 (73.53)	424 (75.18)	112 (67.88)		
Ever	193 (26.47)	140 (24.82)	53 (32.12)		
Days before surgery, Mean ± SD	8.18 ± 4.57	8.32 ± 4.25	7.70 ± 5.51	t=1.338	0.182
Statins, n (%)				χ^2=0.120	0.729
No	590 (80.93)	458 (81.21)	132 (80.00)		
Yes	139 (19.07)	106 (18.79)	33 (20.00)		
β-blockers, n (%)				χ^2=0.845	0.358
No	564 (77.37)	432 (76.60)	132 (80.00)		
Yes	165 (22.63)	132 (23.40)	33 (20.00)		
Calcium channel blockers, n (%)				χ^2=0.312	0.577
No	512 (70.23)	399 (70.74)	113 (68.48)		
Yes	217 (29.77)	165 (29.26)	52 (31.52)		
ACEIs, n (%)				χ^2=0.516	0.472
No	690 (94.65)	532 (94.33)	158 (95.76)		
Yes	39 (5.35)	32 (5.67)	7 (4.24)		
ARBs, n (%)				χ^2=1.603	0.205
No	561 (76.95)	428 (75.89)	133 (80.61)		
Yes	168 (23.05)	136 (24.11)	32 (19.39)		
Diuretics, n (%)				χ^2=0.134	0.714
No	612 (83.95)	475 (84.22)	137 (83.03)		
Yes	117 (16.05)	89 (15.78)	28 (16.97)		
NSAIDs, n (%)				χ^2=2.012	0.156
No	589 (80.8)	462 (81.91)	127 (76.97)		
Yes	140 (19.2)	102 (18.09)	38 (23.03)		
LVEF (%), Mean ± SD	57.85 ± 9.33	58.74 ± 8.43	54.79 ± 11.43	t=4.125	**<.001**
eGFR (ml/min), Mean ± SD	86.01 ± 31.84	88.34 ± 32.43	78.05 ± 28.37	t=3.683	**<.001**
ALT (μ/L), Mean ± SD	28.06 ± 24.10	28.77 ± 24.89	25.64 ± 21.09	t=1.468	0.142
AST (μ/L), Mean ± SD	29.21 ± 23.36	28.82 ± 22.43	30.55 ± 26.34	t=-0.834	0.405
TG (mmol/l), Mean ± SD	1.37 ± 0.72	1.37 ± 0.72	1.33 ± 0.72	t=0.616	0.538
CHOL (mmol/l), Mean ± SD	4.05 ± 1.18	4.08 ± 1.16	3.95 ± 1.24	t=1.257	0.209
HDL (mmol/l), Mean ± SD	1.04 ± 0.28	1.05 ± 0.27	1.01 ± 0.30	t=1.387	0.166
LDL (mmol/l), Mean ± SD	2.56 ± 0.80	2.56 ± 0.77	2.55 ± 0.89	t=0.145	0.884
TB (μmol/L), Mean ± SD	15.40 ± 8.08	15.12 ± 7.90	16.38 ± 8.65	t=-1.760	0.079

DBIL (μmol/L), Mean ± SD	5.67 ± 4.04	5.47 ± 3.84	6.32 ± 4.62	t=-2.150	**0.033**
IBIL (μmol/L), Mean ± SD	9.73 ± 4.71	9.65 ± 4.58	10.04 ± 5.13	t=-0.946	0.344
WBC (10^9/L), Mean ± SD	6.60 ± 2.73	6.44 ± 2.57	7.12 ± 3.16	t=-2.524	**0.012**
NT-ProBNP (pg/ml), M (Q_1, Q_3)	714.20 (249.20, 1424.70)	671.55 (235.95, 1335.80)	950.60 (381.80, 2173.50)	Z=3.898	**<.001**
**Intraoperative**					
Procedure, n (%)	1 (0.14)	1 (0.18)	0 (0.00)	-	**<.001**
Isolated					
Valve replacement	315 (43.21)	255 (45.21)	60 (36.36)		
CABG (off-pump)	151 (20.71)	119 (21.10)	32 (19.39)		
CABG (on-pump)	46 (6.31)	35 (6.21)	11 (6.67)		
Combined	52 (7.13)	33 (5.85)	19 (11.52)		
Ascending aortic aneurysm or dissection surgery	97 (13.31)	58 (10.28)	39 (23.64)		
VSD or ASD repair	37 (5.08)	36 (6.38)	1 (0.61)		
Other	30 (4.12)	27 (4.79)	3 (1.82)		
Duration of surgery (h), Mean ± SD	5.93 ± 1.67	5.77 ± 1.56	6.51 ± 1.88	t=-4.610	**<.001**
Duration of CPB (h), Mean ± SD	2.07 ± 1.35	1.99 ± 1.30	2.36 ± 1.47	t=-3.119	**0.002**
Duration of Clamp (min), Mean ± SD	84.52 ± 63.28	81.81 ± 61.26	93.78 ± 69.17	t=-2.005	**0.046**
Circulatory arrest, n (%)				χ^2=24.555	**<.001**
No	657 (90.12)	525 (93.09)	132 (80.00)		
Yes	72 (9.88)	39 (6.91)	33 (20.00)		
MAP (mmHg), Mean ± SD	68.10 ± 7.59	68.35 ± 7.76	67.27 ± 6.92	t=1.703	0.090
Hb (g/l), Mean ± SD	9.25 ± 1.69	9.34 ± 1.75	8.94 ± 1.43	t=2.996	**0.003**
Red blood cell transfusion (u), M (Q_1, Q_3)	0.00 (0.00, 2.00)	0.00 (0.00, 2.00)	0.00 (0.00, 4.00)	Z=-3.873	**<.001**
Medication Administration					
Dexmedetomidine continuous infusion (ml/h), M (Q_1, Q_3)	0.00 (0.00, 0.00)	0.00 (0.00, 0.00)	0.00 (0.00, 2.00)	Z=-3.346	**<.001**
Propofol injection (mg), M (Q_1, Q_3)	0.00 (0.00, 0.00)	0.00 (0.00, 0.00)	0.00 (0.00, 0.00)	Z=-1.637	0.102
Aminocaprotic acid injection (mg), M (Q_1, Q_3)	0.00 (0.00, 0.00)	0.00 (0.00, 0.00)	0.00 (0.00, 0.00)	Z=-1.797	0.072
Prothrombin complex concentrate infusion (IU), M (Q_1, Q_3)	800.00 (0.00, 800.00)	400.00 (0.00, 800.00)	800.00 (0.00, 800.00)	Z=-4.257	**<.001**
Fibrinogen infusion (g), M (Q_1, Q_3)	1.00 (0.00, 2.00)	0.00 (0.00, 2.00)	2.00 (0.00, 2.00)	Z=-5.560	**<.001**
**Postoperative**					

Lac (mmol/l), Mean ± SD	3.13 ± 2.39	3.00 ± 2.25	3.61 ± 2.77	t=-2.588	**0.010**

SD: standard deviation, M: Median, Q_1: 1st Quartile, Q_3: 3rd Quartile

t: t-test, Z: Mann-Whitney test, χ^2: Chi-square test, -: Fisher exact
