# Supplementary material for: A nomogram for predicting the risk of postoperative delirium in individuals undergoing cardiovascular surgery
Source: Eur J Neurol. 2024 Sep 25;31(12):e16483. doi: 10.1111/ene.16483 (PMC11555157; doi:10.1111/ene.16483)
Supplement: Supplementary file 2 — Table S2. [file ENE-31-e16483-s002.pdf]

Supplementary table 2 Equilibrium test between training and testing set

| Variable | Total (n = 729) | Data<br>train (n = 513) | <br>test (n = 216) | Statistic | *P* |
|---|---|---|---|---|---|
| delirium, n (%) | | | | $\chi^2$=0.134 | 0.714 |
| No | 564 (77.37) | 169 (78.24) | 395 (77.00) | | |
| Yes | 165 (22.63) | 47 (21.76) | 118 (23.00) | | |
| **Preoperative** | | | | | |
| Sex, n (%) | | | | $\chi^2$=0.069 | 0.792 |
| Male | 434 (59.53) | 127 (58.80) | 307 (59.84) | | |
| Female | 295 (40.47) | 89 (41.20) | 206 (40.16) | | |
| Age (y), Mean ± SD | 59.61 ± 11.49 | 59.78 ± 11.37 | 59.54 ± 11.54 | t=0.251 | 0.802 |
| BMI (kg/m$^2$), Mean ± SD | 24.29 ± 3.39 | 24.47 ± 3.55 | 24.21 ± 3.31 | t=0.936 | 0.350 |
| Education, n (%) | | | | $\chi^2$=3.568 | 0.613 |
| Illiteracy | 160 (21.95) | 53 (24.54) | 107 (20.86) | | |
| Primary school | 142 (19.48) | 41 (18.98) | 101 (19.69) | | |
| Junior middle school | 243 (33.33) | 69 (31.94) | 174 (33.92) | | |
| High school | 106 (14.54) | 26 (12.04) | 80 (15.59) | | |
| College | 37 (5.08) | 12 (5.56) | 25 (4.87) | | |
| University and above | 41 (5.62) | 15 (6.94) | 26 (5.07) | | |
| Smoking, n (%) | | | | $\chi^2$=0.885 | 0.642 |
| Never | 451 (61.87) | 128 (59.26) | 323 (62.96) | | |
| Yes | 171 (23.46) | 54 (25.00) | 117 (22.81) | | |
| Ever | 107 (14.68) | 34 (15.74) | 73 (14.23) | | |
| Drinking, n (%) | | | | $\chi^2$=0.277 | 0.599 |
| Never | 566 (77.64) | 165 (76.39) | 401 (78.17) | | |
| Yes | 163 (22.36) | 51 (23.61) | 112 (21.83) | | |
| Hypertension, n (%) | | | | $\chi^2$=0.180 | 0.671 |
| No | 248 (34.02) | 71 (32.87) | 177 (34.50) | | |
| Yes | 481 (65.98) | 145 (67.13) | 336 (65.50) | | |
| Diabetes, n (%) | | | | $\chi^2$=0.139 | 0.709 |
| No | 581 (79.7) | 174 (80.56) | 407 (79.34) | | |
| Yes | 148 (20.3) | 42 (19.44) | 106 (20.66) | | |
| Asthma, n (%) | | | | $\chi^2$=1.199 | 0.274 |
| No | 712 (97.67) | 213 (98.61) | 499 (97.27) | | |
| Yes | 17 (2.33) | 3 (1.39) | 14 (2.73) | | |
| Malignancy, n (%) | | | | $\chi^2$=0.284 | 0.594 |
| No | 709 (97.26) | 209 (96.76) | 500 (97.47) | | |
| Yes | 20 (2.74) | 7 (3.24) | 13 (2.53) | | |
| ASA, n (%) | | | | $\chi^2$=3.620 | 0.164 |
| II+III | 496 (68.04) | 140 (64.81) | 356 (69.40) | | |
| IV | 180 (24.69) | 63 (29.17) | 117 (22.81) | | |
| IIIE+IVE | 53 (7.27) | 13 (6.02) | 40 (7.80) | | |

| | | | | | |
|---|---|---|---|---|---|
| Cardiac surgery, n (%) | | | | $\chi^2$=0.688 | 0.407 |
| Never | 710 (97.39) | 212 (98.15) | 498 (97.08) | | |
| Ever | 19 (2.61) | 4 (1.85) | 15 (2.92) | | |
| PCI, n (%) | | | | $\chi^2$=0.549 | 0.459 |
| Never | 695 (95.34) | 204 (94.44) | 491 (95.71) | | |
| Ever | 34 (4.66) | 12 (5.56) | 22 (4.29) | | |
| CVA, n (%) | | | | $\chi^2$=1.435 | 0.231 |
| Never | 631 (86.56) | 192 (88.89) | 439 (85.58) | | |
| Ever | 98 (13.44) | 24 (11.11) | 74 (14.42) | | |
| TIA, n (%) | | | | $\chi^2$=0.772 | 0.380 |
| Never | 698 (95.75) | 209 (96.76) | 489 (95.32) | | |
| Ever | 31 (4.25) | 7 (3.24) | 24 (4.68) | | |
| AF, n (%) | | | | $\chi^2$=0.268 | 0.605 |
| Never | 536 (73.53) | 156 (72.22) | 380 (74.07) | | |
| Ever | 193 (26.47) | 60 (27.78) | 133 (25.93) | | |
| Days before surgery, Mean ± SD | 8.18 ± 4.57 | 8.00 ± 4.48 | 8.26 ± 4.61 | t=-0.701 | 0.483 |
| Statins, n (%) | | | | $\chi^2$=1.442 | 0.230 |
| No | 590 (80.93) | 169 (78.24) | 421 (82.07) | | |
| Yes | 139 (19.07) | 47 (21.76) | 92 (17.93) | | |
| β-blockers, n (%) | | | | $\chi^2$=1.403 | 0.236 |
| No | 564 (77.37) | 161 (74.54) | 403 (78.56) | | |
| Yes | 165 (22.63) | 55 (25.46) | 110 (21.44) | | |
| Calcium channel blockers, n (%) | | | | $\chi^2$=2.384 | 0.123 |
| No | 512 (70.23) | 143 (66.20) | 369 (71.93) | | |
| Yes | 217 (29.77) | 73 (33.80) | 144 (28.07) | | |
| ACEI, n (%) | | | | $\chi^2$=0.271 | 0.603 |
| No | 690 (94.65) | 203 (93.98) | 487 (94.93) | | |
| Yes | 39 (5.35) | 13 (6.02) | 26 (5.07) | | |
| ARBs, n (%) | | | | $\chi^2$=1.935 | 0.164 |
| No | 561 (76.95) | 159 (73.61) | 402 (78.36) | | |
| Yes | 168 (23.05) | 57 (26.39) | 111 (21.64) | | |
| Diuretics, n (%) | | | | $\chi^2$=0.266 | 0.606 |
| No | 612 (83.95) | 179 (82.87) | 433 (84.41) | | |
| Yes | 117 (16.05) | 37 (17.13) | 80 (15.59) | | |
| NSAIDs, n (%) | | | | $\chi^2$=1.802 | 0.180 |
| No | 589 (80.8) | 168 (77.78) | 421 (82.07) | | |
| Yes | 140 (19.2) | 48 (22.22) | 92 (17.93) | | |
| LVEF (%), Mean ± SD | 57.85 ± 9.33 | 8.51 ± 9.07 | 57.57 ± 9.44 | t=1.246 | 0.213 |
| eGFR (ml/min), Mean ± SD | 86.01 ± 31.84 | 87.81 ± 32.83 | 85.25 ± 31.41 | t=0.994 | 0.320 |
| ALT (μ/L), Mean ± SD | 28.06 ± 24.10 | 26.39 ± 18.96 | 28.77 ± 25.95 | t=-1.214 | 0.225 |
| AST (μ/L), Mean ± SD | 29.21 ± 23.36 | 27.03 ± 15.43 | 30.13 ± 25.95 | t=-1.639 | 0.102 |
| TG (mmol/L), Mean ± SD | 1.37 ± 0.72 | 1.41 ± 0.79 | 1.35 ± 0.70 | t=0.963 | 0.336 |
| CHOL (mmol/L), Mean ± SD | 4.05 ± 1.18 | 4.04 ± 1.11 | 4.06 ± 1.21 | t=-0.206 | 0.837 |
| HDL (mmol/L), Mean ± SD | 1.04 ± 0.28 | 1.03 ± 0.27 | 1.04 ± 0.28 | t=-0.679 | 0.497 |

| | | | | | |
|---|---|---|---|---|---|
| LDL (mmol/L), Mean ± SD | 2.56 ± 0.80 | 2.56 ± 0.83 | 2.56 ± 0.78 | t=0.106 | 0.915 |
| TB (μmol/L), Mean ± SD | 15.40 ± 8.08 | 15.21 ± 7.04 | 15.49 ± 8.49 | t=-0.417 | 0.677 |
| DBIL (μmol/L), Mean ± SD | 5.67 ± 4.04 | 5.54 ± 3.09 | 5.72 ± 4.38 | t=-0.529 | 0.597 |
| IBIL (μmol/L), Mean ± SD | 9.73 ± 4.71 | 9.67 ± 4.55 | 9.76 ± 4.78 | t=-0.250 | 0.803 |
| WBC ($10^9$/L), Mean ± SD | 6.60 ± 2.73 | 6.52 ± 2.58 | 6.63 ± 2.79 | t=-0.517 | 0.606 |
| NT-ProBNP (pg/ml), M ($Q_1$, $Q_3$) | 714.20 (249.20, 1424.70) | 695.75 (237.45, 1326.05) | 715.60 (269.10, 1482.20) | Z=-0.427 | 0.669 |
| **Intraoperative** | | | | | |
| Procedure, n (%) | 1 (0.14) | 0 (0.00) | 1 (0.19) | - | 0.437 |
| Isolated | | | | | |
| Valve replacement | 315 (43.21) | 98 (45.37) | 217 (42.30) | | |
| CABG (off-pump) | 151 (20.71) | 43 (19.91) | 108 (21.05) | | |
| CABG (on-pump) | 46 (6.31) | 15 (6.94) | 31 (6.04) | | |
| Combined | 52 (7.13) | 20 (9.26) | 32 (6.24) | | |
| Ascending aortic aneurysm or dissection surgery | 97 (13.31) | 23 (10.65) | 74 (14.42) | | |
| VSD or ASD repair | 37 (5.08) | 7 (3.24) | 30 (5.85) | | |
| Other | 30 (4.12) | 10 (4.63) | 20 (3.90) | | |
| Duration of surgery (h), Mean ± SD | 5.93 ± 1.67 | 5.84 ± 1.60 | 5.97 ± 1.69 | t=-1.016 | 0.310 |
| Duration of CPB (h), Mean ± SD | 2.07 ± 1.35 | 2.06 ± 1.31 | 2.07 ± 1.37 | t=-0.147 | 0.883 |
| Duration of Clamp (min), Mean ± SD | 84.52 ± 63.28 | 82.69 ± 61.49 | 85.29 ± 64.07 | t=-0.506 | 0.613 |
| Circulatory arrest, n (%) | | | | $\chi^2$=0.402 | 0.526 |
| No | 657 (90.12) | 197 (91.20) | 460 (89.67) | | |
| Yes | 72 (9.88) | 19 (8.80) | 53 (10.33) | | |
| MAP (mmHg), Mean ± SD | 68.10 ± 7.59 | 68.62 ± 7.28 | 67.88 ± 7.71 | t=1.206 | 0.228 |
| Hb (g/l), Mean ± SD | 9.25 ± 1.69 | 9.19 ± 1.66 | 9.28 ± 1.71 | t=-0.590 | 0.556 |
| Red blood cell transfusion (u), M ($Q_1$, $Q_3$) | 0.00 (0.00, 2.00) | 0.00 (0.00, 2.00) | 0.00 (0.00, 2.00) | Z=-1.020 | 0.308 |
| Medication Administration | | | | | |
| Dexmedetomidine continuous infusion (ml/h), M ($Q_1$, $Q_3$) | 0.00 (0.00, 0.00) | 0.00 (0.00, 0.50) | 0.00 (0.00, 0.00) | Z=-0.733 | 0.464 |
| Propofol injection (mg), M ($Q_1$, $Q_3$) | 0.00 (0.00, 0.00) | 0.00 (0.00, 0.00) | 0.00 (0.00, 0.00) | Z=-0.170 | 0.865 |
| Aminocaprotic acid injection (mg), M ($Q_1$, $Q_3$) | 0.00 (0.00, 0.00) | 0.00 (0.00, 0.00) | 0.00 (0.00, 0.00) | Z=-0.049 | 0.961 |
| Prothrombin complex concentrate infusion (IU), M ($Q_1$, $Q_3$) | 800.00 (0.00, 800.00) | 800.00 (0.00, 800.00) | 800.00 (0.00, 800.00) | Z=-0.094 | 0.925 |
| Fibrinogen infusion (g), M ($Q_1$, $Q_3$) | 1.00 (0.00, 2.00) | 1.00 (0.00, 2.00) | 1.00 (0.00, 2.00) | Z=-0.282 | 0.778 |
| **Postoperative** | | | | | |

| | | | | | |
|---|---|---|---|---|---|
| Lac (mmol/l), Mean ± SD | 3.13 ± 2.39 | 2.91 ± 2.17 | 3.23 ± 2.48 | t=-1.668 | 0.096 |

SD: standard deviation, M: Median, $Q_1$: 1st Quartile, $Q_3$: 3rd Quartile

t: t-test, Z: Mann-Whitney test, $\chi^2$: Chi-square test, -: Fisher exact
